# Factors Influencing Intraocular Pressure Changes after Laser In Situ Keratomileusis with Flaps Created by Femtosecond Laser or Mechanical Microkeratome

**DOI:** 10.1371/journal.pone.0147699

**Published:** 2016-01-29

**Authors:** Meng-Yin Lin, David C. K. Chang, Yun-Dun Shen, Yen-Kuang Lin, Chang-Ping Lin, I-Jong Wang

**Affiliations:** 1 Department of Ophthalmology, Shuang Ho Hospital, Taipei Medical School of Medicine, Taipei Medical University, Taipei County, Taiwan; 2 Institute of Clinical Medicine, School of Medicine, National Taiwan University, Taipei, Taiwan; 3 Nobel Eye Clinic, Taipei, Taiwan; 4 Biostatistics Center, Taipei Medical University, Taipei, Taiwan; 5 Department of Ophthalmology, National Taiwan University Hospital, Taipei, Taiwan; 6 Institute of Clinical Medicine, School of Medicine, China Medical University, Taichung, Taiwan; National Eye Institute, UNITED STATES

## Abstract

The aim of this study is to describe factors that influence the measured intraocular pressure (IOP) change and to develop a predictive model after myopic laser in situ keratomileusis (LASIK) with a femtosecond (FS) laser or a microkeratome (MK). We retrospectively reviewed preoperative, intraoperative, and 12-month postoperative medical records in 2485 eyes of 1309 patients who underwent LASIK with an FS laser or an MK for myopia and myopic astigmatism. Data were extracted, such as preoperative age, sex, IOP, manifest spherical equivalent (MSE), central corneal keratometry (CCK), central corneal thickness (CCT), and intended flap thickness and postoperative IOP (postIOP) at 1, 6 and 12 months. Linear mixed model (LMM) and multivariate linear regression (MLR) method were used for data analysis. In both models, the preoperative CCT and ablation depth had significant effects on predicting IOP changes in the FS and MK groups. The intended flap thickness was a significant predictor only in the FS laser group (*P* < .0001 in both models). In the FS group, LMM and MLR could respectively explain 47.00% and 18.91% of the variation of postoperative IOP underestimation (R^2^ = 0.47 and R^2^ = 0.1891). In the MK group, LMM and MLR could explain 37.79% and 19.13% of the variation of IOP underestimation (R^2^ = 0.3779 and 0.1913 respectively). The best-fit model for prediction of IOP changes was the LMM in LASIK with an FS laser.

## Introduction

Laser in situ keratomileusis (LASIK) is the most popular corneal refractive surgical procedure for myopia, hyperopia, and astigmatism corrections in this decade [1]. In this procedure, corneal flaps are created and lifted to expose the corneal stroma for ablation. The method for flap creation has evolved from a mechanical microkeratome (MK) to a femtosecond (FS) laser over these years in consideration of safety, particularly for patients with thin corneas or small orbits [2]. Moreover, with a superior performance in visual quality, the FS laser has gained popularity [3, 4].

Patients with myopia have higher risk of glaucoma [5]. LASIK surgery involves flap dissection and central corneal thickness (CCT) reduction, which subsequently cause underestimation of the postoperative IOP (postIOP) [6]. Moreover, after LASIK, topical steroid is usually used to reduce postoperative inflammation, which might predispose patients to IOP elevation and glaucoma [7]. If we don’t know the normal range of postIOP, the iatrogenic low IOP might delay early detection of steroid responders or glaucoma.

Modern tonometry techniques such as pressure phosphene tonometry [8], rebound tonometry [9], dynamic contour tonometry [10], and ocular response analysis (ORA) have been employed to obviate IOP underestimation after LASIK. However, noncontact tonometry (NCT) remains the most widely used technique because it has a low cost, is easy to use, exhibits a high correlation with the Goldmann applanation tonometer (GAT) [11], and involves no direct contact with corneal flaps. We therefore developed the statistical models of NCT, which might be helpful for early detection of ocular hypertension and delineate the interplay among factors to determine the IOP change after LASIK surgery with an FS laser or MK.

Previous studies have reported that preoperative age, IOP, CCT, and MSE are factors influencing IOP underestimation after LASIK performed using an MK [12–14]. Some studies demonstrated that the flap dissection also influences the IOP change after LASIK surgery [15–17]. Besides, the flap created by an FS laser has a planar configuration and better thickness predictability than that of a traditional MK [18, 19]. Hence, we assumed that the flap difference would be significant in predicting the IOP change, and compared the prediction of IOP change after LASIK surgery with an FS laser or MK. The purpose of this study was to establish the statistical models for estimating the IOP change, according to NCT in patients undergoing LASIK for myopia and myopic astigmatism. In addition, we added flap thickness as an explanatory covariate and compared the accuracy among different statistical models and different methods of flap dissection.

## Patients and Methods

In this retrospective study, we reviewed the medical records of patients who underwent myopic LASIK, with flaps created using an FS laser or an MK, from August 2006 to June 2014 at the Department of Ophthalmology, National Taiwan University and Nobel Clinic, Taipei, Taiwan. The research protocol was approved by the Human Research and Ethics Committee of National Taiwan University Hospital. The patient records/information was anonymized and de-identified prior to analysis. The minimum age of the patients was 21 years, and they were considered suitable for LASIK only after detailed screening examinations. Patients were excluded if they had a history of an ocular disease, trauma, surgery, diabetes mellitus, or other systemic diseases known to affect the eyes. Patients who developed a new ocular illness that interfered with the outcomes during the follow-up were excluded from the study.

### Surgical techniques

Two experienced surgeons (D.C.K.C. and I.J.W.) performed all LASIK procedures. Bladeless flaps were created using a 60-kHz IntraLase FS laser (AMO, Abbott Park, Illinois). All flaps had a superior hinge, and the intended thickness ranged from 100 to 120 μm. The raster line and spot separation were 8 μm. The raster energy was either 1.0 or 1.2 mJ, and the sidecut energy was 1.4 mJ. The MK flaps were created using Moria M2 (Moria, Antony, France) with a superior hinge, and the intended thicknesses were 110 μm and 130 μm. All stromal beds were ablated using a Star S4 excimer laser (AMO, Abbott Park, Illinois, USA). Emmetropia was attempted in all cases by using an ablation zone ranging from 6.5 × 6.5 to 6.5 × 5.0 mm for spherical and astigmatic corrections, respectively. The blend zone diameter was 8.0 mm. The postoperative topical medication regimen consisted of ciprofloxacin 4 times per day, Tobradex (Alcon, Fort Worth, Texas, USA) every 2 hours, and artificial tears (Refresh; Allergan, Irvine, CA, USA) every hour for 1 day. From the second day, the 3 eye drops were administered 4 times per day for 1 week, and 0.1% Fluorometholone (Oasis, Taiwan) was subsequently administered 4 times per day at a dose that tapered to the end of the month.

### Outcome measures

Both eyes were recruited in patients who underwent bilateral LASIK. The preoperative variables were age, sex, MSE, ablation depth, CCT (measured using Sonomed 200PC, Sonomed, Inc., New Berlin, WI, USA), CCK (measured using Topcon KR 8100, Ijssel, Netherlands), IOP, and flap thickness. The flap thickness was recorded as the intended flap setting in the surgery. After the operation, the patients were followed up at 1 week and at 1, 6, and 12 months. Patients who attended follow-up at 1 week but did not return for follow-up at 1 month were excluded.

### Intraocular pressure measurement

All IOP values were measured using NCT (Topcon CT 60 computerized tonometer; Topcon, Tokyo, Japan). IOP measurements were first reviewed a month before LASIK, and then a day before the surgery; and at 1 week and 1, 6, and 12 months after LASIK. At each visit, IOP was examined 3 times in each eye. The mean IOP obtained during averaged IOP measurements was used in the analysis.

### Statistical analysis

To compare the predictability of IOP change (postIOP–preIOP), we applied two statistical methods to analyze the effects of these preoperative parameters. In the multivariate linear regression method (MLR), since two eyes of one subject are highly dependent, we randomly selected one eye from one subject. In the linear mixed model (LMM), since it can manage the dependent data [20], both eyes of one subject were included.

The variables which were proved to be significant in previous studies, such as preoperative MSE, ablation depth, IOP, age, sex, CCK, and CCT, were taken into consideration. As the major differences between the FS laser and the MK lie in flap morphology and predictability of thickness, the flap thickness was also included as the variable. To avoid multicollinearity, the correlations between any two variables were analyzed by Pearson correlation. At each follow-up, at least three IOP values were obtained for each eye and the serial measurements were averaged to represent the IOP at that time point. The mean IOPs of all preoperative visits were averaged again to obtain the mean preoperative IOP.

The accuracy of our models was checked by the goodness-of-fit statistic pseudo R2 with a least-squares method. We calculated the squares of the difference between the observed and the predicted values of IOP changes. The residual sum of squares were estimated as the unexplained proportion of IOP variation.

$${\text{R}}^{2}=\left(1\;-\;\text{residual sum of squares}/\text{total variation}\right)=\text{the explained proportion by the model}$$

All statistical analyses were performed using SAS 9.3 (Cary, NC, USA).

## Results

We retrospectively reviewed preoperative, intraoperative, and 12-month postoperative medical records for 2485 eyes of 1309 patients who underwent LASIK for myopia and myopic astigmatism. After excluding patients who did not return for follow-up 1 month after the operation and the ones with missing data, we evaluated 1228 eyes of 685 patients in the FS laser group and 704 eyes of 355 patients in the MK group. While selecting variables, we observed a highly negative correlation between preoperative MSE and ablation depth (correlation coefficient = -0.86). Therefore, we only included the ablation depth for analysis. The other variables exhibited low correlations among each other (all *P* < .001). The correlation coefficient between flap thickness and CCT was 0.30, and the correlation coefficient between flap thickness and MSE was −0.12. Ultimately, age, sex, ablation depth, flap thickness, CCT, and CCK were processed for further analysis.

**Fig 1:**
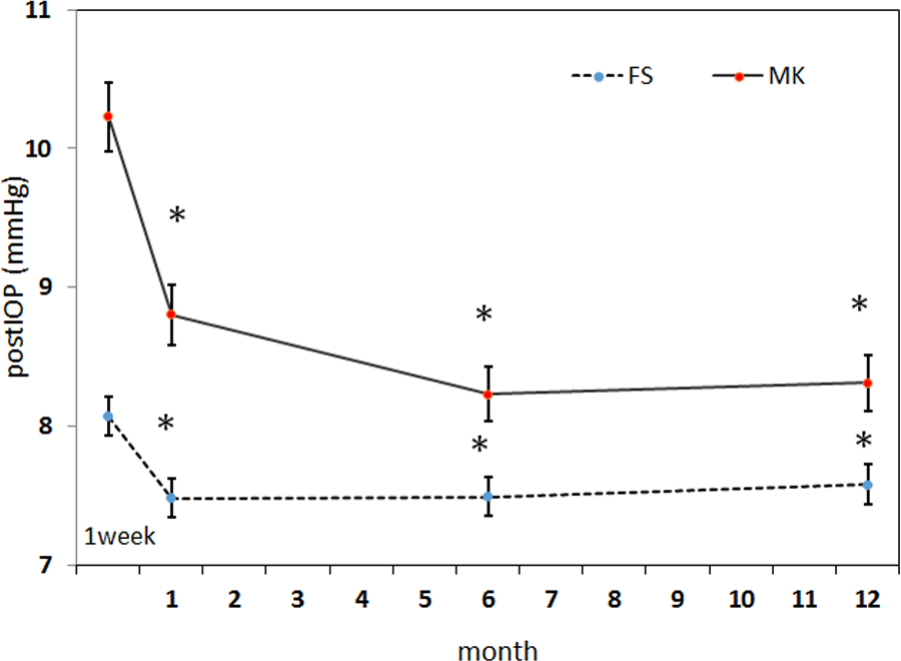
The mean IOP.after LASIK using an FS laser or MK. Data are presented as mean ± standard deviation at 1 week, 1 month, 6 months and 12 months after surgery. In both methods of flap dissection, the postIOP at 1 month, 6 months and 12 months were all significantly lower than postIOP at 1 week (the * indicates *P*< .0001). The MK group showed greater postIOP from 1 week to 1 month than those of the FS laser group.

Table 1 lists the descriptive data of FS and MK groups. The mean age was 31.6 ± 6.0 years in the FS laser group and 29.8 ± 5.7 years in the MK group. The average MSE was −6.0 ± 1.6 diopters (D) in the FS laser group and −5.6 ± 1.9 D in the MK group. The mean CCT was 535.1 ± 34.6 and 549.2 ± 34.0 μm in the FS laser and MK groups, respectively. The mean ablation depth was 87.5 ± 21.7 μm in the FS group and 82.8 ± 24.8 μm in the MK group. The mean intended flap thickness was 103.9 ± 6.1 and 126.4 ± 8.2 μm in the FS laser and MK groups, respectively. In the FS laser group, the intended flap thickness ranged from 100 to 120 μm; 62.59% of the eyes had an intended flap thickness of 100 μm, 34.47% had an intended flap thickness of 110 μm, and only 2.62% had an intended flap thickness of 120 μm. In the MK group, 78.67% of eyes had an intended flap thickness of 130 μm, and 21.33% had an intended flap thickness of 100 μm. The mean preIOP was 14.2 ± 3.2 mmHg in the FS laser group; 15.5 ± 2.9 mmHg in the MK group. Fig 1 showed the mean postIOP in both groups. In FS laser group, the postIOP at 1 week was 8.07 ± 2.49 mmHg; at 1 month was 7.48 ± 2.42 mmHg; at 6 months was 7.49 ± 2.4 mmHg; at 12 months was 7.56 ± 2.46 mmHg. In the MK group, the postIOP at 1 week was 10.23 ± 2.88 mmHg; at 1 month was 8.80 ± 2.45 mmHg; at 6 months was 8.23 ± 2.41 mmHg; at 12 months was 8.31 ± 2.51 mmHg. In both groups, the postIOP at 1 week were significantly higher than postIOP at 1 month, 6 months and 12 months (all *P*< .0001). Accordingly, we included the postIOP at 1, 6 and 12 months for prediction of IOP change.

**Table 1:** Clinical characteristics of the FS laser and MK groups.

Variable	FS laser group (*n* = 1,228 eyes)	MK group (*n* = 704 eyes)	*P* value§
Number of patients	685	355	−
Age at operation (years)	31.6 ± 6.0	29.8 ± 5.7	< .001
MeanK (diopter)	43.7 ± 1.4	43.7 ± 1.3	0.842
CCT (μm)	535.1 ± 34.6	549.2 ± 34.0	< .001
Flap thickness (μm)	103.9 ± 6.1	126.4 ± 8.2	< .001
Ablation depth	87.5 ± 21.7	82.8 ± 24.8	< .001
MSE (diopter)	−6.0 ± 1.6	−5.6 ± 1.9	< .001
PreIOP (mmHg)	14.2 ± 3.2	15.5 ± 2.9	< .001
PostIOP (mmHg)	7.8 ± 2.5	8.9 ± 2.4	< .001

§ *t* test.

Table 2 showed the factors influencing IOP changes in subjects undergoing LASIK with an FS laser. The significant predictors in the FS group were CCT, ablation depth and flap thickness (all *P*< .0001 in both MLR and LMM). A goodness-of-fit R^2^ correlation coefficient was 0.47 in the LMM (*P*< .0001) and 0.19 in the MLR (*P*< .0001). Table 3 showed the LMM and MLR model of LASIK with an MK. The significant predictors were ablation depth (*P* < .0001 in both models) and CCT (*P* = 0.1 in the LMM; *P* = 0.035 in the MLR model). The flap thickness, however, was insignificant in the MK group in both statistical models (*P* = 0.94 in the LMM; *P* = 0.82 in MLR). The CCK was also an insignificant predictor in both FS laser and MK group. For the MK group, the R^2^ values were 0.38 in the LMM (*P*< .0001) and 0.19 in the MLR model (*P*< .0001). Using the LMM, each micron increase of ablation depth would result in 0.035 mmHg of IOP reduction in the FS laser group and 0.034 mmHg of IOP reduction in the MK group; each micron increase of flap thickness in FS laser group, would result in 0.043mmHg of IOP reduction.

**Table 2:** Association of clinical characteristics with IOP change in patients undergoing LASIK with an FS laser determined using an LMM and MLR.

	Linear mixed model			Multiple linear regression		
Variable	Estimate	*S*.*E*.	*P*	Estimate	*S*.*E*.	*P*
Intercept	17.788	3.6	< .0001	16.515	2.081	0.002
Age at OP (β_1_)	-0.010	0.016	0.519	-0.008	0.014	0.586
gender^‡^ (β_2_)	-0.461	0.219	0.036	-0.219	0.124	0.078
MeanK (β_3_)	-0.1	0.0687	0.147	-0.470	0.189	0.013
CCT (β_4_)	-0.012	0.003	< .0001	-0.089	0.060	0.14
Flap thickness(β_5_)	-0.045	0.007	< .0001	-0.042	0.006	< .0001
Ablation depth (β_6_)	-0.035	0.004	< .0001	-0.036	0.004	< .0001

‡When the patient is male, gender = 1; otherwise gender = 0; PreIOP = preoperative IOP; CCT = preoperative central corneal thickness; Age at OP = age at operation

**Table 3:** Association of clinical characteristics with IOP change in patients undergoing LASIK with an MK determined using an LMM and MLR.

	Linear mixed model			Multiple linear regression		
Variable	Estimate	*S*.*E*.	*P*	Estimate	*S*.*E*.	*P*
Intercept	0.508	5.121	0.921	1.692	4.532	0.709
Age at OP(β_1_)	0.011	0.023	0.636	0.007	0.02	0.734
gender^‡^ (β_2_)	0.058	0.304	0.848	-0.109	0.265	0.68
Mean K (β_3_)	-0.024	0.095	0.805	-0.020	0.084	0.813
CCT (β_4_)	-0.006	0.004	0.099	0.003	-2.12	0.034
Flap thickness (β_5_)	0.001	0.016	0.942	-0.003	0.015	0.818
Ablation depth (β_6_)	-0.038	0.005	< .0001	-0.039	0.005	< .0001

‡When the patient is male, gender = 1; otherwise gender = 0; PreIOP = preoperative IOP; CCT = preoperative central corneal thickness; Age at OP = age at operation

**Fig 2:**
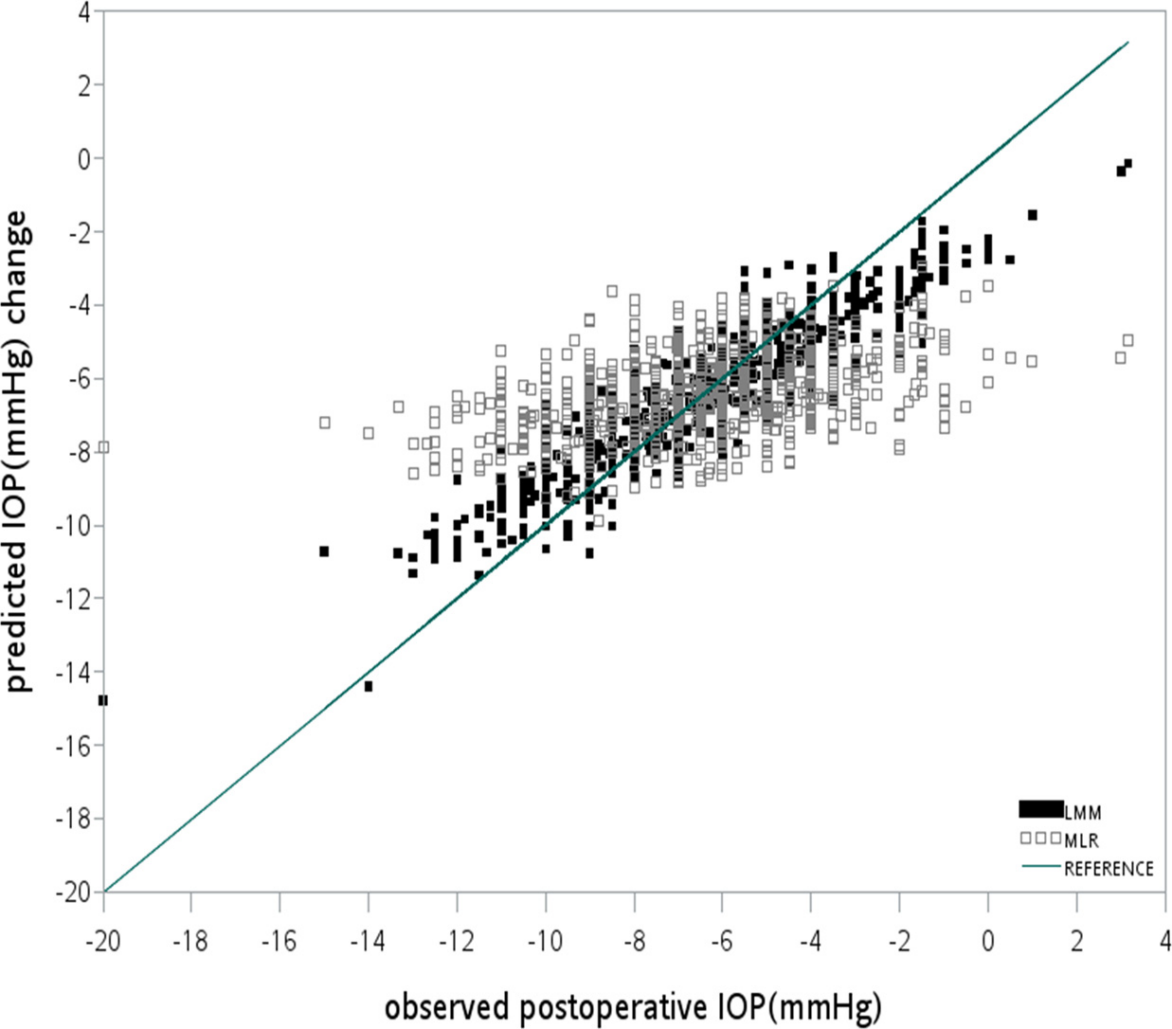
Distribution of the observed IOP changes and that predicted by LLM and MLR in LASIK with an FS laser. The reference line indicates the ideal condition, in which the predicted IOP changes equal the observed IOP changes. The LMM (R^2^ = 0.47) had superior prediction accuracy compared with the MLR model (R^2^ = 0.1891).

**Fig 3:**
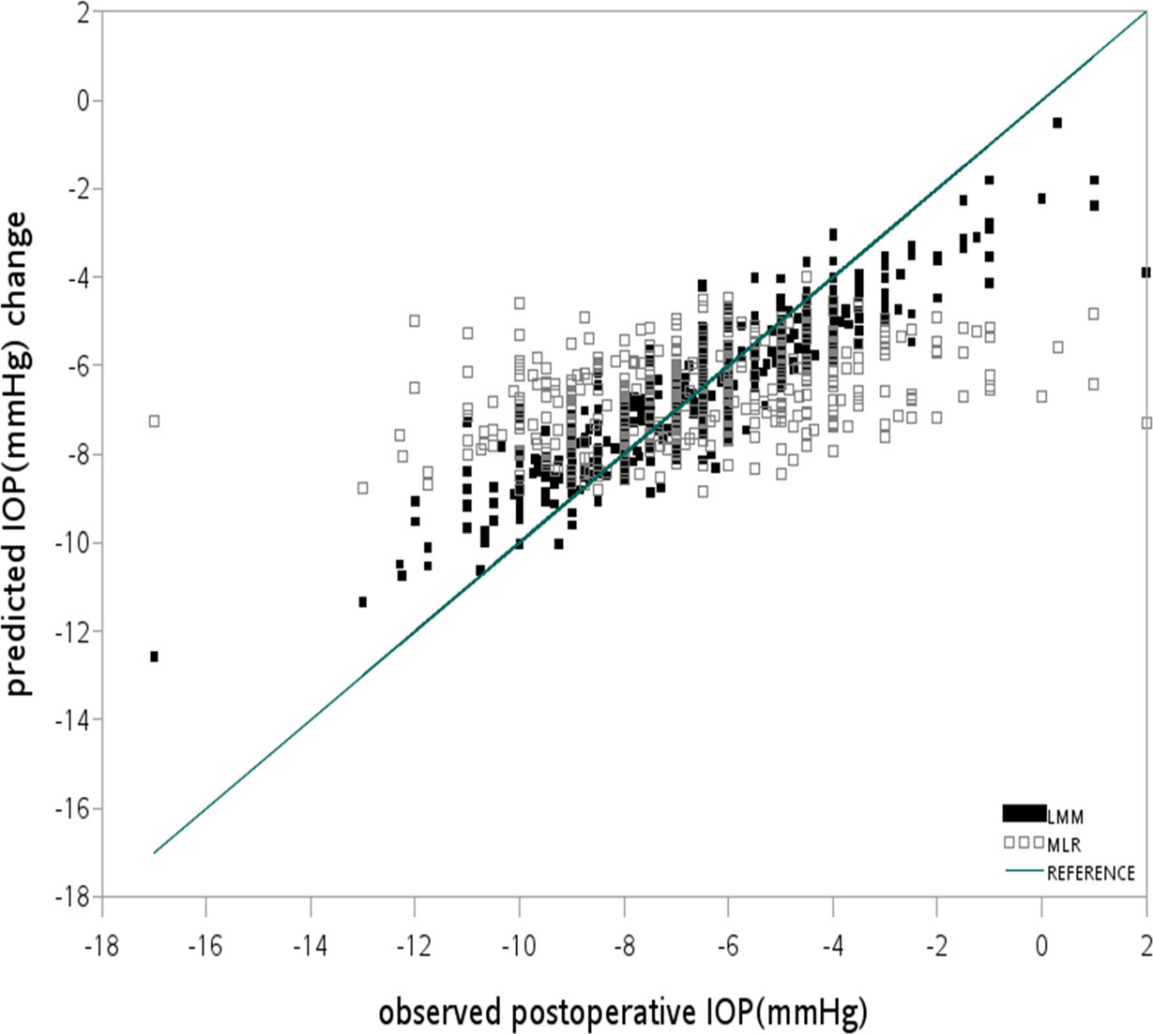
Distribution of the observed IOP changes and that predicted by LLM and MLR in LASIK with an MK. The reference line indicates the ideal condition, in which the observed IOP changes equal the predicted IOP changes. The LMM (R^2^ = 0.3779) had superior prediction accuracy compared with the MLR model (R^2^ = 0.1913).

Figs 2 and 3 showed the scatter plots of the observed and predicted IOP change (postIOP–preIOP) of LASIK with an FS laser and MK respectively. The reference line indicates the ideal condition when the predicted IOP change equals the observed IOP change. In both figures, the distribution of the dots predicted by the LMM was closer to the reference line than those of the MLR when either an FS laser or an MK was used. This indicates that the prediction accuracy of the LMM was superior to that of MLR.

## Discussion

Previous studies have discussed the IOP change after LASIK with an MK. For example, Chang et al used univariate and multivariate linear regression to demonstrate IOP change after LASIK performed using an automated corneal shaper (ACS, Chiron, Emeryville, CA, USA) or a Hansatome MK (Bausch & Lomb, Rochester, NY, USA)[15]. Their best-fit formula showed that the IOP change was a function of the refractive change. They attributed the intercept value as the IOP reduction caused by flap dissection, which was approximately 1.36 mmHg. Sanchez-Naves et al used a similar concept and found that flap cutting using a Moria M2 (Moria, Antony, France) 130 microkeratome resulted in an IOP reduction of approximately 1.6 ± 0.8 mmHg [16]. In both studies, IOP reduction after LASIK was assumed as a function of the refractive error [15] or ablation depth [16]. Kohlhaas et al used MLR to analyze the IOP change measured by GAT after LASIK with MK [21]. They viewed IOP change as a function of (540-CCT) and (43-CCK). Here, 540 and 43 were considered the physiological values of CCT and CCK. The intercept value of 0.75 mmHg was then attributed to decreased corneal stability. In brief, instead of individual flap parameters, the intercept value was considered the average IOP reduction from flap dissection in the aforementioned studies.

To the best of our knowledge, this is the first study, which compared the IOP reduction between LASIK using an MK or FS. We observed that CCT and ablation depth were significant in predicting IOP change in the MK group; CCT, ablation depth and flap thickness were significant predictors in the FS group. The intended flap thickness created with the FS laser was correlated with significant IOP reduction, but, in contrast, flap thickness was not a significant predictor in the MK group. This might be attributed to the higher precision of flap thickness using an FS laser and the greater variability of flap thickness created by an MK [22–25]. This result was also validated with higher prediction in the FS group than that in the MK group. In the LMM, R^2^ was 0.47 in the FS group; R^2^ was 0.37 in the MK group.

The LMM is a statistical model containing fixed and random effects. It is particularly useful in settings where repeated measurements are obtained in a longitudinal study or where measurements are made on clusters of related statistical units [20]. In the present study, most patients underwent operation for both eyes so the dependency between both eyes cannot be overlooked. Hence we included both eyes in the LMM. As to the MLR method, which is not designed for the correlated data, it is not logical to include both eyes to build up a model to predict the IOP change of one eye. We therefore selected only one eye from each subject in the MLR method.

Yang et al used an LMM to examine the relationship between preoperative parameters and postIOP [26]. They found that preoperative IOP, CCT, MSE, ablation depth and gender were significant variables in predicting postIOP and the R^2^ was 0.91. Schallhorn et al also used the LMM to examine IOP change after PRK or LASIK using an FS or MK in myopic or hyperopic correction in a substantial number of patients [17]. They regarded the intercept value (0.94 mmHg) as the average IOP reduction contributed by flap dissection in LASIK surgery. The R^2^ value was 0.45 in the myopic LASIK group. After we changed the outcome variable to the IOP changes, the R^2^ value becomes as 0.47, which is comparable to that of Schallhorn et al [17].

In the present study, in the LMM, for each micron increase of flap thickness, the postIOP decreased by 0.045 mmHg; for each micron increase in the ablation depth, the postIOP decreased by 0.035 mmHg. This means that the effect of flap dissection per micron on IOP reduction was slightly stronger than that of stromal ablation. This might be partly due to the larger diameter of the flap than the stromal ablation. In addition, Winkler et al found that high degree of interconnectivity of transverse fibers is most pronounced in the anterior stroma, which is essential in stabilizing cornea [27]. Santhiago et al also investigated the relative contribution of flap thickness and ablation depth to the percentage of tissue altered in ectasia after LASIK [28]. They concluded that flap thickness is more relevant as a risk factor for developing ectasia than the stromal ablation. These findings were consistent with our statistical results.

The ideal method to measure postIOP would be a device that is free of corneal factors. GAT is the current gold standard, but its accuracy is still affected by corneal thickness [29]. Several studies have still shown the IOP reduction after LASIK with GAT [14, 30]. Although the reading of NCT is dependent on CCT, and may be more sensitive to changes in CCT than GAT [31], it remains the most widely used instrument because it has low cost, ease to use, and no direct contact with corneal flaps. In addition, a large meta-analysis has demonstrated that NCT has good agreement with GAT [32]. Therefore, statistical models for prediction of the postIOP change of NCT still have their value for clinical application. Moreover, the statistical model facilitates delineating the interplay of various factors in determining IOP change. With our models, we can predict IOP change and detect ocular hypertension early in patients undergoing LASIK surgery.

To overcome the influence of the corneal thickness on the measured postIOP, new tonometers, such as ORA (Ocular Response Analyzer, Reichert Ophthalmic Instruments, Buffalo, NY) or Corvis®ST (Oculus Optikgeräte GmbH, Wetzlar, Germany) tonometers, were developed to measure the IOP through the corneal biomechanical properties. The ORA is a dynamic instrument, which is affected by elastic and viscous properties of corneas [33]. The corneal compensated IOP is able to negate corneal properties partially, but it still measures a reduced IOP [33, 34]. Corvis®ST tonometer is a novel noncontact tonometer, which records dynamic deformation of the cornea to calculate the IOP value [35]. Hong et al compared postIOP with GAT, ORA and Corvis®ST tonometers. They found that Corvis®ST tonometer obtained higher IOP values than other tonometry techniques [36]. Unfortunately, the prices of both tonometers are still too expensive to be used for our daily practice.

The superiority of our study lies in several aspects. First, we investigated the effects of FS and MK flaps respectively. Since flap morphology and predictability of these 2 methods are different, their contribution to IOP changes might be different. Second, instead of regarding the intercept value as the effect of flap dissection, we specifically used flap thickness as a covariate in our model, which let us quantify the effect of flap dissection. Third, we did not include the IOP changes at 1 week in the prediction model. It had been demonstrated that postIOP at 1 week are usually higher than the other time points. Since the application of the prediction model would mostly be several months or years after LASIK, exclusion of postIOP at 1 week would lessen the bias of IOP prediction. Fourth, we followed the IOP changes for up to 12 months, at which the postIOP are more stable and comparable to the IOP thereafter.

This study also had some limitations. First, we used the intended flap thickness rather than the intraoperative flap thickness. The highly variable flap thickness in the MK group would impede the accuracy of our prediction. Second, we recruited only patients with conventional ablation settings, with the ablation zone ranging from 6.5 × 6.5 to 6.5 × 5.0 mm for spherical and astigmatic corrections, respectively. We did not recruit patients who underwent multizone or wavefront laser ablation. This would restrict the application to other different ablation settings. Third, our models were based on the assumption that the absolute IOP remains constant after operation. If the underlying conditions changed because of trauma or other ocular illnesses, the absolute IOP might change, and our predicted IOP change and the consequent postIOP would not be the standard for ocular hypertension. Nonetheless, our models are valid in most circumstances in which the IOP was measured using NCT before and after LASIK. Fourth, we measured IOP with NCT [31]. However, NCT has been shown to have good agreement with GAT.[33] It would be interesting to have other types of tonometry against which to evaluate the noncontact results, particularly dynamic contour tonometry, which may be less influenced by CCT [37]. The corneal biomechanics after LASIK was affected by flap thickness, flap configuration, stromal ablation depth, and interlamellar healing [38]. Therefore, change in the corneal biomechanics would in turn influence postIOP measurement. These influences on postIOP exhibit interindividual variation because of factors such as surgeon preference and instrumental factors such as the types of MK and FS laser. Our statistical model can be used to explain the biomechanical effects of preoperative CCK, CCT, age, ablation depth, and flap thickness on IOP changes after LASIK surgery.

In conclusion, we applied a large sample size to evaluate factors influencing IOP changes obtained using NCT after LASIK performed using an FS laser or MK. Flap dissection by using an FS laser caused significant IOP reduction, but the effect was nonsignificant when an MK was used. This difference might be attributed to the higher variability in flap thickness in the MK group, when compared with that of the FS group. Including flap thickness as a covariate increases the accuracy of our prediction, and implies the importance of the method of flap dissection in IOP reduction after LASIK surgery.

## What Was Known

The preoperative CCT, MSE, and IOP had significant effects on predicting postIOP in LASIK with the femtosecond laser and microkeratome. The impact of flap dissection per micron on IOP change after LASIK surgery was lower than the stromal ablation per micron.

## What This Paper Adds

The best-fit model for IOP prediction was the linear mixed model for LASIK using femtosecond laser. The significant predictors included preoperative CCT, MSE, IOP and the intended flap thickness in LASIK with femtosecond laser. The impact of flap dissection per micron on IOP change after LASIK surgery was stronger than the stromal ablation per micron.
